# Metformin-Enhanced Digital Therapeutics for the Affordable Primary Prevention of Diabetes and Cardiovascular Diseases: Advancing Low-Cost Solutions for Lifestyle-Related Chronic Disorders

**DOI:** 10.3390/healthcare13243220

**Published:** 2025-12-09

**Authors:** Brian Farley, Emi Radetich, Joseph DAlessandro, Grzegorz Bulaj

**Affiliations:** 1 College of Pharmacy, University of Utah, Salt Lake City, UT 84112, USA; 2 Cadence Health Advisors, Salt Lake City, UT 84106, USA; 3 Department of Medicinal Chemistry, College of Pharmacy, University of Utah, Salt Lake City, UT 84112, USA

**Keywords:** mobile app, digital therapeutic, insulin, healthcare costs, healthcare insurance, nutrition, health coaching, AI, pharmacy care, obesity, risk factors

## Abstract

Each year, over 1 million people in the United States die from diabetes and cardiovascular diseases (CVDs). These largely preventable chronic conditions also create a financial burden on patients, payers, and healthcare systems. The popularity of GLP-1-based management of cardiometabolic conditions can escalate healthcare spending, while incentivizing digitization of semaglutide (Ozempic, Wegovy), tirzepatide (Mounjaro), and others using the “prescription drug use-related software” (PDURS) framework. In this article, we highlight opportunities to advance digital-first interventions and metformin-enhanced digital therapeutics (DTx) for the primary prevention of diabetes and CVDs. Metformin is a low-cost antidiabetic medication that is effective in preventing diabetes and cardiovascular adverse events. Concurrently, digital health technologies for managing chronic conditions, e.g., Dario Health, Omada Health, and WellDoc, enable digital-first and drug + digital combination therapies for prediabetes and those at risk for CVDs. We describe incentives for advancing Affordable Primary Prevention (APP), suggesting that nonprofit healthcare systems, such as Kaiser Permanente, Intermountain Health or Ascension Health, payers such as Cigna and Aetna/CVS Health, or private equity investors can leverage their venture funds to support development of metformin-enhanced DTx. In conclusion, (1) the PDURS framework can accelerate innovation of preventive medicine by bridging precision digital interventions with low-cost generic drugs, and (2) integrating healthy behaviors with pharmacotherapies is essential for the financially sustainable prevention of lifestyle-related chronic diseases.

## 1. Introduction

**Figure 1:**
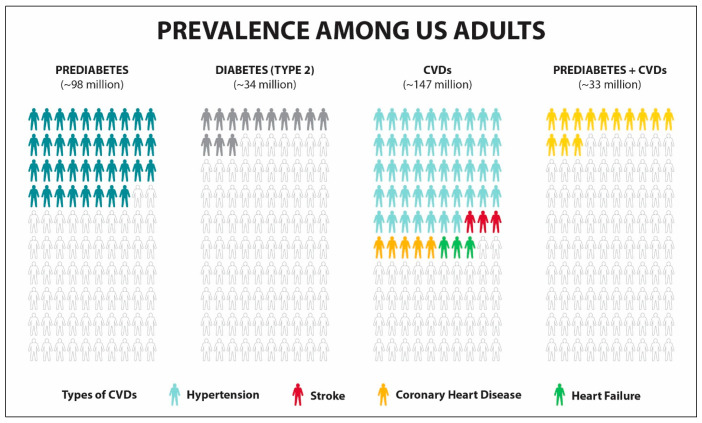
Prevalence of prediabetes, diabetes (type 2), and cardiovascular diseases among adults in the US. Numbers in parenthesis reflect estimated number of people living with a specific chronic condition. Prediabetes, T2DM, and CVD prevalence estimated using 2024 data.

Chronic diseases are a crisis within the United States (US) [1,2]. These conditions are defined as diseases that last one year or longer and require ongoing treatment to improve symptoms and health-related quality of life (HRQoL). The most prevalent chronic diseases that are the leading causes of death and disability in the US are cardiovascular diseases (CVDs), type 2 diabetes mellitus (T2DM), and cancer. CVDs comprise such conditions as hypertension, coronary heart disease, stroke, and heart failure. Over 940,000 Americans died from CVD-related causes in 2022 and over 100,000 from diabetes [3]. As illustrated in Figure 1, among adults in the US, the prevalence of prediabetes is 38%, T2DM is 13%, CVDs is 68%, and combined prediabetes and CVDs is 13% [4,5,6]. The types of CVDs can be broken down into hypertension at 47%, stroke at 3%, coronary heart disease 5%, and heart failure at 3% [3]. It is noteworthy that despite launching the National Diabetes Prevention Program (DPP) in 2010, prevalence of diabetes increased by 18% between 2012 and 2020 [6,7]. Despite reporting positive outcomes of this lifestyle intervention in preventing T2DM, its long-term effectiveness remains unknown [7,8]. Furthermore, over a period of eight years, only approx. 5% of people at risk for diabetes enrolled in the National DPP, highlighting the need for scaling preventive interventions [9]. The prevalence of cardiovascular conditions such as hypertension is also raising [10]. The prevalence of cardiometabolic conditions in the US is expected to increase through 2050 [11].

Patients living with CVDs and diabetes report a lower quality of life for them and their families, in part due to the decreased quality of care they experience. Those with T2DM can experience a variety of symptoms ranging from gastrointestinal and neuropathy to bone infection, sight loss, and/or kidney failure [12,13]. As for CVDs, symptoms can range from feeling out of breath despite lack of movement to heart injury or attack. As a result, patients are at a much higher risk of comorbid depression and anxiety, which further compounds the burden added to the healthcare system [14]. In addition to patients, healthcare providers who treat these patients are also negatively impacted through chronic stress and burnout, leading to decreased quality of care and increased prevalence of medication errors [15].

Chronic diseases are not only emotionally costly, but financially costly as well. For patients with T2DM, in 2017, the average amount of money spent on healthcare by a single person was USD 16,750, which is more than twice as much spent on healthcare than those who did not have T2DM [16]. A major driver of this cost is insulin [17,18,19]. Meanwhile, patients with CVDs spent approximately USD 4423 annually on healthcare on average between 2014 and 2018 [20]. The financial impact can be further illustrated by the rise of relatively novel medications, such as GLP-1 receptor agonists (GLP1RAs) [21]. While GLP1RAs, such as semaglutide, dulaglutide, and tirzepatide, are effective in the reduction in A1c and weight, they do not always demonstrate cost-effectiveness [22,23]. From 2018 to 2023 alone, the total spending on GLP1RAs increased by 500%, escalating financial burdens for patients and payers [24]. Economic burden of diabetes in the US is captured in the following American Diabetes Association (ADA) statement: “The total estimated cost of diagnosed diabetes in the U.S. in 2022 is $412.9 billion, including $306.6 billion in direct medical costs and $106.3 billion in indirect costs attributable to diabetes” [25]. Similarly, “adult cardiovascular spending increased from $212 billion in 1996 to $320 billion in 2016, a period when the US population increased by >52 million people, and median age increased from 33.2 to 36.9 years. Over this period, public insurance was responsible for the majority of cardiovascular spending (54%), followed by private insurance (37%) and out-of-pocket spending (9%)” [26]. Driven mostly by chronic diseases, healthcare spending in the US exceeded USD 5 trillion in 2024 [27,28].

To simultaneously reduce the prevalence and economic costs of chronic diseases, there are needs to innovate low-cost solutions that combine affordable pharmacy care and preventive medicine at scale. To this end, a novel framework of prescription drug use-related software (PDURS) offers integration of pharmacotherapies with behavior change. In this perspective article, we highlight an opportunity to develop PDURS-based primary prevention of diabetes and CVDs through the integration of metformin coupled with lifestyle interventions delivered through digital therapeutics (DTx).

## 2. Chronic Disease Prevention

**Figure 2:**
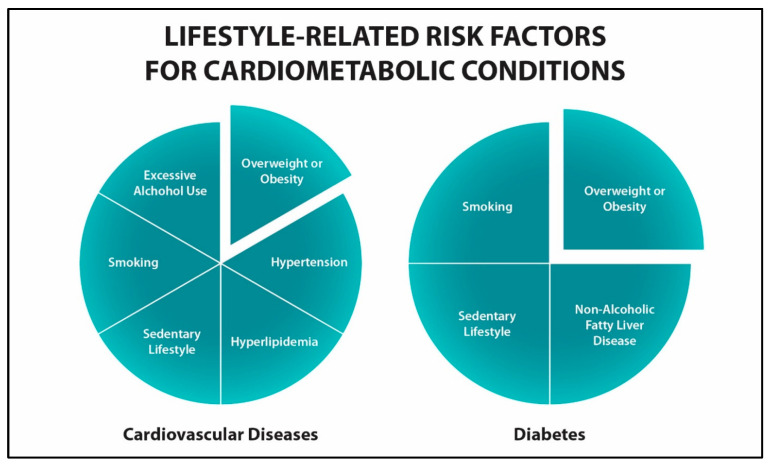
Examples of modifiable risk factors for diabetes and cardiovascular diseases.

*Lifestyle-based prevention*: As illustrated in Figure 2, there are a variety of preventable risk factors that contribute to diabetes and CVDs [29,30,31]. Lifestyle interventions, such as dietary modification and increased physical activity, have proven to be effective in preventing and treating cardiometabolic conditions [32,33,34]. These interventions are commonly performed via educational handouts and classes, motivational interviewing, referrals to dieticians, and frequent follow-ups. Coordinated care models that integrate blood sugar, blood pressure, and cholesterol management have shown promise in improving cardiovascular outcomes among patients with T2DM and established CVD [35]. The ADA emphasizes aggressive risk factor modification as a cornerstone of CVD prevention in T2DM, with evidence suggesting improved 10-year coronary heart disease risk and declining ASCVD-related morbidity and mortality [36]. Despite these advances, translating metabolic improvements into long-term cardiovascular benefits remains a key challenge in preventive care.

Many of these same interventions for T2DM affect those who solely have CVD. The American Heart Association (AHA) defines these as “Life’s Essential 8” [37]. These eight metrics determine the quality of one’s cardiovascular health: diet, physical activity, nicotine exposure, sleep health, BMI, blood lipids, blood glucose, and blood pressure. The AHA also emphasizes that various factors affect these metrics, such as favorable social determinants of health. People are more likely to have better cardiovascular health if they also have higher income, education, employment status, appropriate socialization, and/or less racial discrimination and incarceration [38]. As a result, equity is a huge consideration when assessing the types of interventions available to a person to improve their cardiovascular health. There are many ways these lifestyle interventions can be communicated to the population. For example, public health campaigns can raise awareness for patients seeking diagnosis for CVD and T2DM. However, these campaigns have more benefit for these one-time interventions and less benefit for sustainable habitual changes due to continuous competition with the commercial determinants of health [39].

*Pharmacy-based prevention*: While lifestyle decisions and behaviors can prevent disease, additional support via pharmacy care can also benefit patients and people at risk for chronic conditions. In a 2025 meta-analysis comparing the all-cause mortality and CVD outcomes with a variety of primary prevention interventions, medications that lowered blood pressure were statistically significant for reducing the risk of a CVD-related mortality by 21% [40]. As for the studies that measured time to major cardiovascular event (CVE), blood-pressure-lowering medications were also found to be statistically significant for lowering the risk of a major CVE by 16% [40]. The most prevalent antihypertensives that lowered these risks were ACE inhibitors and beta-blockers [40]. While aspirin is the FDA-approved drug for the secondary prevention of cardiovascular events, however its use for the primary prevention is controversial due to the GI bleeding risks [41]. Given their broad effectiveness and popularity, there is an interest in using GLP1RAs for prevention of diabetes and cardiovascular conditions [42,43,44].

While GLP1RAs have received worldwide attention for their impact on cardiometabolic conditions, metformin remains a mainstay of therapy for the treatment of T2DM [45]. Metformin is a prescription medication widely used for diabetes management that has a potential for preventing diabetes and CVDs. Metformin lowers blood glucose by inhibiting hepatic gluconeogenesis, decreasing intestinal absorption, and increasing peripheral glucose uptake to improve insulin sensitivity. It also has a favorable adverse effect profile, as patients most commonly experience gastrointestinal (GI) upset in the form of nausea, bloating, diarrhea, and abdominal cramping. GI upset typically occurs at the introduction of treatment, which is prevented by slow titration of the dose and managed by taking metformin with food. Also, metformin has been associated with vitamin B12 deficiency due to malabsorption [46]. While this is a concern, since vitamin B12 deficiency can cause neuropathy, this deficiency can be managed with supplementation and nutritional interventions, which can be addressed within the digital therapeutic with dietary guidance to eat more low-fat animal products and fortified foods [47]. In patients who develop renal dysfunction, metformin can accumulate and cause lactic acidosis; however, it does not cause outright renal dysfunction nor lactic acidosis on its own [48,49]. It is recommended to monitor the safety of metformin in people with chronic kidney disease (CKD) using serum creatinine and the estimated glomerular filtration rate (eGFR) [50].

With its efficacy in the treatment of T2DM and its favorable safety profile, the use of metformin to prevent T2DM in patients diagnosed with prediabetes has high potential. The US Diabetes Prevention Program performed a long-term randomized, controlled trial comparing metformin versus placebo with lifestyle intervention versus placebo for the outcome of T2DM incidence. The study was conducted from 1996 to 2020, resulting in a 21-year follow-up of the 3195 participants who had prediabetes. Compared to placebo, lifestyle interventions reduced incidence of T2DM by 24% and metformin reduced incidence by 17% [51]. In a different systematic review and meta-analysis, lifestyle interventions reduced the incidence of T2DM by 25% compared with metformin [52]. When metformin and lifestyle interventions are combined, a multicenter randomized, controlled trial found that the combination lowered T2DM incidence 17% more than lifestyle interventions alone. This study also noted that there was no increase in serious adverse events, and that most patients reported GI upset with metformin [53]. A different meta-analysis also found that while lifestyle modification decreased the incidence of T2DM by 58% compared to the control group, metformin decreased the incidence by 31% compared to the placebo group in most individuals [54]. In obese individuals, metformin decreased incidence of T2DM by 53% compared to lifestyle modifications by 51%. In young adults (age 25–44), metformin decreased incidence by 44% compared to lifestyle modifications by 48%. These findings show that metformin is almost as effective as lifestyle modifications in preventing diabetes, supporting evaluations of combining lifestyle modifications and metformin for improved effectiveness.

The evidence does not just suggest that metformin decreases the incidence of T2DM in obese individuals; metformin can also decrease the burden of obesity itself with modest weight loss. In the same US Diabetes Prevention Program RCT that suggested metformin can prevent the progression of prediabetes into T2DM, a post hoc analysis was performed on weight loss [55]. Among the patients who had sustained weight loss of greater than or equal to 5% after one year, the participants who were randomized to metformin achieved sustained weight loss in years 6–15 of follow-up. In a different meta-analysis of 49 RCTs in patients who did not have T2DM, the effect of metformin on weight loss was analyzed compared to placebo/control, lifestyle interventions, and FDA-approved weight loss medications [45]. While metformin did not have greater reduction in BMI than lifestyle interventions or weight loss medications, it did have statistically significant decrease in BMI compared to placebo/control, with a mean difference [MD] of −0.56 [−0.74, −0.37] kg/m^2^ at doses between 500 mg and 2500 mg per day. At doses of 1700 mg per day, the percentage change in BMI was −2.53% [−2.90, −2.17]. While not significantly different than lifestyle changes alone, these results can be extrapolated to suggest that metformin combined with lifestyle interventions can augment weight loss. Metformin-augmented lifestyle changes would be guided in the PDURS-based primary prevention.

While there used to be a school of thought that metformin does not have an impact on cardiovascular event reduction, when a meta-analysis of randomized, controlled trials analyzed a subset of RCTs that do not include other anti-glycemic agents, metformin was found to reduce all-cause mortality and major adverse cardiovascular events (MACEs) by 21% compared to placebo/no therapy [56]. A recent meta-analysis of thirteen studies (including nine observational cohorts, three randomized controlled trials, and one nested case–control study) found that, relative to other active glucose-lowering therapies, metformin use was associated with significantly lower risks of all-cause mortality (pooled RR 0.71 [0.61–0.84]) and cardiovascular events (pooled RR 0.76 [0.60–0.97]) among individuals with T2DM and mild-to-moderate chronic kidney disease [57]. In other studies, the effectiveness of metformin in lowering CVD incidence seemed to improve over time, mainly when used in younger populations [58]. Table 1 highlights the key similarities and differences between metformin and GLP1RA.

## 3. Digital Health Technologies for Diabetes and CVDs

Digital health technologies belong to a broad category of software- and device-based solutions related to monitoring, treating, and preventing medical conditions. They comprise mobile apps, video games, wearables, and other devices intended to improve health and therapy outcomes. Digital therapeutics belong to a group of digital health technologies known as Software as a Medical Device (SaMD) products [88,89]. DTx can be marketed as Prescription Digital Therapeutics (PDTs), over-the-counter (OTC) DTx, or under “reinforcement discretion” for specific indications based on the FDA authorization using several regulatory pathways. The recently proposed Composite Digital Therapeutics Index (cDTI) offers a means to evaluate the efficacy, safety, engagement, and quality of clinical evidence for PDTs [90]. DTx are recognized as tools to improve chronic disease management, including diabetes and other cardiometabolic conditions [91,92,93,94].

Research studies support the use of digital health technologies for the management of diabetes and CVDs [95,96,97]. Furthermore, these technologies are also recognized for their use in prevention of diabetes and CVDs [98,99,100,101]. Table 2 provides few examples of available digital health platforms and mobile apps for cardiometabolic conditions. One of the first mobile apps that received the FDA marketing authorization for diabetes is BlueStar, pioneered by digital health company Welldoc (Columbia, MD, USA). Currently, Welldoc offers AI-powered platform that integrates real-time personalized coaching for patients with healthcare professionals and health plans. Other digital health platforms offer health monitoring, coaching, and education coupled with virtual care. Digital health solutions for prediabetes and diabetes also include mobile apps combined with wearable continuous glucose monitoring (CGM) systems [102]. Technologies like Dexcom G7 and Stelo (Dexcom), Lingo and FreeStyle Libre or Rio (Abbott), and Simplera Sync (Medtronic) are available either on prescription or over the counter, and offer real-time tracking of blood glucose levels while offering feedback insights and lifestyle management tools. Advances in personalized AI health agents offer more tools to optimize real-world and just-in-time adaptive interventions for prevention and management of chronic conditions, including diabetes and CVDs [98,103,104].

Digital health technologies have unique abilities to target sedentary lifestyle and sleep hygiene through direct-to-consumer wearable devices such as Apple Watch, Galaxy Watch, Garmin Watch, FitBit, Oura ring, and others [105,106]. These tools, coupled with mobile apps, translate biometric data into feedback and actionable health-related insights. When worn continuously, they measure sleep quality, heart rate and heart rate variability, stress levels, and burnt calories, as well as diverse types of physical activities and exercises [107]. These wearable technologies have the abilities to promote behavior change by empowering users towards improving their sleep quality, stress management, and physical exercise. Research studies support the use of fitness and sleep trackers to promote lifestyle modifications among people living with prediabetes, diabetes, and CVDs [107,108,109,110,111]. Wearables are also recognized as remote-monitoring tools that can be integrated with behavior change interventions for the primary prevention of diabetes and CVDs [112,113]. Notably, recent integration of Oura’s sleep and activity tracker with Stelo’s CGM illustrates prospects for increasing the precision prevention of chronic conditions. In addition to lifestyle-promoting wearables, there are also technologies focused on reducing the major risk factors for diabetes and CVDs. For smoking cessation, examples of digital health technologies include smartphone apps such as Clickotine, QuitGuide, and Quit Journey, as well as QuitBot, an AI-powered chatbot that uses cognitive behavioral therapy to help nicotine users quit their addiction [114,115,116].

## 4. Integrating Digital Health and Pharmaceutical Drugs

**Figure 3:**
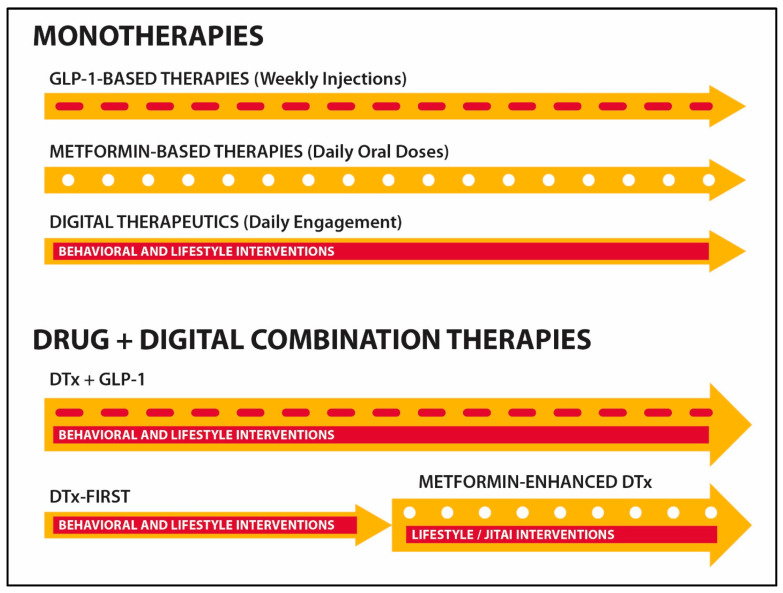
Comparing drug- and DTx-based monotherapies with drug + digital combination therapies that integrate pharmacological and non-pharmacological modalities. Drug + digital combination therapies can be delivered using adjunctive DTx- or PRURS-based mobile apps, supporting symbiotic relationships between patients and pharmacy care.

In contrast to “drug-alone” treatments, drug + digital combinations therapies can simultaneously deliver pharmacological and behavioral interventions while improving medication adherence, patient engagement, and disease prognosis (Figure 3) [117,118,119]. Early efforts of integrating digital interventions with pharmacotherapies include the Welldoc’s mobile app for diabetes management and Pear Therapeutics’ reSET-O as adjunctive DTx for opioid use disorder in combination with buprenorphine [120,121]. Our early attempts to create drug + digital combination therapy for epilepsy were encouraged by the drug–device combination product strategy (DTx are considered to be Software as a Medical Device products) [122,123]. The development of a mobile medical app CT-152 and its subsequent FDA authorization as adjunctive DTx for major depressive disorder in combination with antidepressant drugs illustrate a regulatory strategy for drug + digital combination therapies (CT-152 is currently marketed as Rejoyn by Otsuka Precision Health) [124]. Companies like Click Therapeutics, Close Loop Medicine, and Remepy develop drug + device combination therapies for diverse chronic conditions.

In 2023, the FDA published draft guidelines on the Prescription Drug Use-Related Software framework that enables integrating drug labeling with mobile apps, including DTx [125]. There are two labeling standards for FDA approval of PDURS applications: (1) “promotional labeling,” which does not state that the software provides clinical benefit, and (2) “FDA-required labeling,” which allows for companies to include the software on the drug label as part of the clinical benefit. The key factors that influence whether the PDURS has promotional or FDA-required labeling are as follows:

1. Does the PDURS provide a function that is essential to the safe and effective use of the product?
2. Is there evidence that supports the clinical benefits of the PDURS?
3. Does the PDURS rely on data directly transferred from the device constituent part of a combination product?

Evidence that supports the clinical benefit of PDURS can help the FDA approve the PDURS with FDA-required labeling. There are not strict standards as to how the studies should be performed, so real-world evidence and clinical evidence could be used to support the clinically meaningful benefits. Based on PDURS-based opportunities to innovate pharmacotherapies, Click Therapeutic develops so called “Software-Enhanced Drugs™” that integrate medications with matching DTx, yielding drug + digital combination therapies.

**Figure 4:**
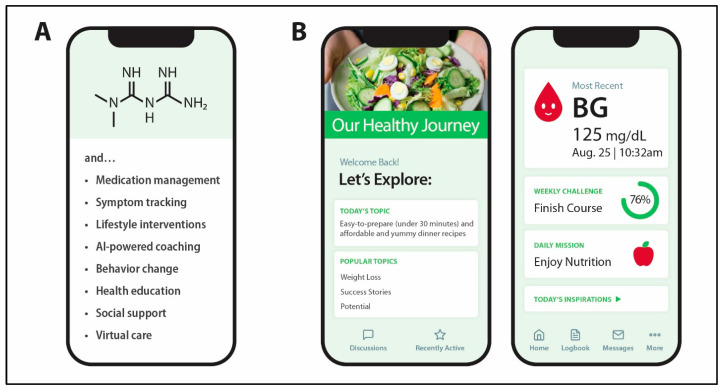
Examples of mobile app features that support “digital-first” and drug + digital combination therapies for the prevention and treatment of cardiometabolic conditions. (**A**)—Digital content of PDURS-based app to improve patient outcomes by integrating behavioral and lifestyle interventions with pharmacotherapies. (**B**)—Examples of mobile app content highlighting opportunities to integrate personalized nutrition and pharmacotherapies.

A combination of digital interventions with metformin or other diabetes drugs is more effective for glycemic control, as compared to drugs alone [126,127]. For cardiometabolic conditions, the clinical and financial successes of semaglutide, dulaglutide, liraglutide, and tirzepatide incentivize pharma and digital health companies to combine GLP1RAs with digital health applications [128,129,130]. As illustrated in Figure 4, medication-coupled mobile apps can improve patient outcomes through diverse features including medication management, symptom tracking and feedback, coaching, education, and promoting healthy nutrition, hence reinforcing behavior change. While cost-effectiveness studies for GLP-1 drugs for diabetes, obesity, and cardiovascular + obesity indications continue to emerge, their high out-of-pocket costs create a significant financial burden on individual patients [22,131,132,133]. Given current challenges of affordable healthcare in the US, the future use of GLP-1 drugs, even when coupled with DTx, for the primary prevention of diabetes and cardiovascular conditions at scale is unlikely to be feasible. As aforementioned, metformin is a low-cost drug that is sparingly used to prevent progression of prediabetes and cardiovascular events [134,135]. To innovate preventive medicine for diabetes and CVDs, herein we also spotlight prospects for developing precision prevention at scale that combines two scenarios: (1) “digital-first” interventions and (2) a combination of “digital-first” and “metformin-enhanced DTx” interventions.

## 5. Low-Cost Primary Prevention of Diabetes and CVDs

Due to low risk and high scalability, digital health interventions offer unprecedented opportunities for the development and large-scale implementation of precision prevention of lifestyle-related chronic conditions. Digital technologies that promote lifestyle interventions, behavior change and health education are well suited not only for the management of diabetes and CVDs, but also to support people who are at risk for these chronic conditions [98]. We recently described the implementation of digital health technologies into retail pharmacies to scale adoption of DTx and wearables that promote lifestyle modifications [136]. Below, we outline prospects for scaling precision prevention using DTx and metformin + DTx combinations.

Traditional primary care is generally defined by in-person appointments to a clinic to see a provider, such as a physician, nurse practitioner, or physician assistant, typically licensed in either family medicine or internal medicine. A virtual care model offers either asynchronous or synchronous telemedicine services. As compared to the traditional model, virtual care model is more scalable, as providers are not limited to appointment schedules and can review patient information in a more flexible manner. DTx have the ability to support lifestyle modifications and behavior change while bridging the gap between visits to a doctor in both the traditional and virtual care models.

**Figure 5:**
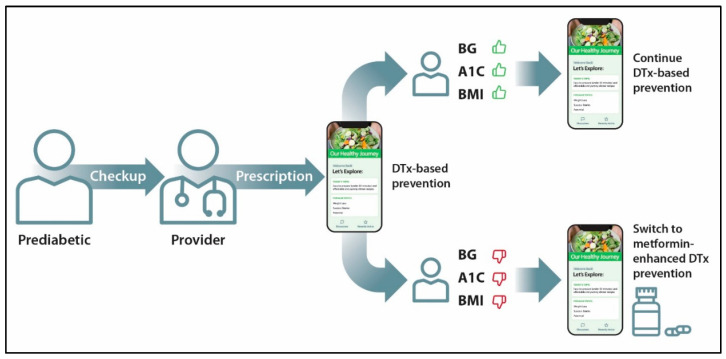
An overview of the user journey with the digital-first and the PDURS-based app plus metformin for the primary prevention of diabetes and CVDs. BG—blood glucose; A1C—hemoglobin A1C biomarker; BMI—body mass index.

One example of DTx-based asynchronous preventive care can be in the form of patient–provider communications related to patient’s lab work. Based on the ADA guidelines, the benchmark indicators for prediabetes are an A1C of 5.7–6.4%, fasting glucose of 100–125 mg/dL, and an OGTT two-hour blood glucose of 140–199 mg/dL (Table 3). Patients with prediabetes, if left untreated, typically progress into type 2 diabetes within 5 years of their diagnosis [137]. The benchmarks for type 2 diabetes are an A1C of 6.5% or higher, a fasting blood glucose of 126 mg/dL or higher, and an OGTT two-hour blood glucose of 200 mg/dL or higher. As shown in Figure 5, upon a patient meeting the criteria for prediabetes, digital health platforms may offer personalized interventions that support physical activities, nutrition, sleep quality, stress management, health education, and other self-management practices. Examples of such “digital-first” interventions are as follows: (1) mobile apps delivering Diabetes Prevention Program [98,138], (2) The Nutritionist Buddy Diabetes app delivering lifestyle interventions [139], and (3) a mobile app Diabetes Risk Score that combines self-assessment and behavior change for people with undiagnosed prediabetes and diabetes [140]. However, there are continuous needs to improve the effectiveness of digital monotherapies for prediabetes [141].

For patients who struggle with controlling prediabetes biomarkers with digital-first interventions, PDURS-based app enables metformin-enhanced digital therapy, hence integrating lifestyle interventions and behavior change with a low-cost prescription drug that is effective in prediabetes (Figure 5). In the PDURS-based APP model, a patient could start on metformin 850 mg, while the app would provide dosing reminders, information on side effect management, exercise plans, diets, continuous glucose monitoring, and an estimated A1C using wearable devices (Figure 4). The doctor, pharmacist, and patient could have access to this information and would be able to collaborate using the app to support the patient’s goals. In this scenario, pharmacists may play a critical role in improving prediabetes outcomes [142,143,144]. Research on progression from normal glucose tolerance to prediabetes suggest that digital-first interventions can also apply to people with pre-prediabetes [145,146].

The aforementioned approach to prediabetes can be applied to prevent CVDs [147,148,149]. Lifestyle interventions show positive effects in preventing CVDs [150,151]. Furthermore, multiple studies suggest the effectiveness of digital health technologies to prevent heart failure, coronary heart disease, and hypertension [152,153,154]. As illustrated in Table 2, the benchmarks of CVDs that could be measured continuously with wearable devices are limited, but would allow for a window into general heart health [99,112]. Patients could also self-monitor blood pressure taken at home to provide feedback and allow for the provider to look for trends during preventive interventions [155]. There is also a well-documented link between prediabetes and CVD, so digital platforms such as Welldoc, Dario Health, Omada Health, and metformin-enhanced DTx interventions could be evaluated for their ability to prevent CVDs [93]. It is noteworthy that the Diabetes Prevention Program Outcomes Study showed that neither metformin (850 mgs twice daily) nor lifestyle interventions could reduce major cardiovascular events, such as myocardial infarction, stroke or cardiovascular death), highlighting needs for long-term studies on combining digital interventions with metformin to prevent CVDs [156,157]. AI-based digital interventions that comprise blood pressure monitoring, activity trackers, and precision lifestyle coaching may improve long-term prevention of cardiovascular conditions [158,159,160].

## 6. Incentives for Innovating the Primary Prevention of Lifestyle-Related Chronic Diseases

**Figure 6:**
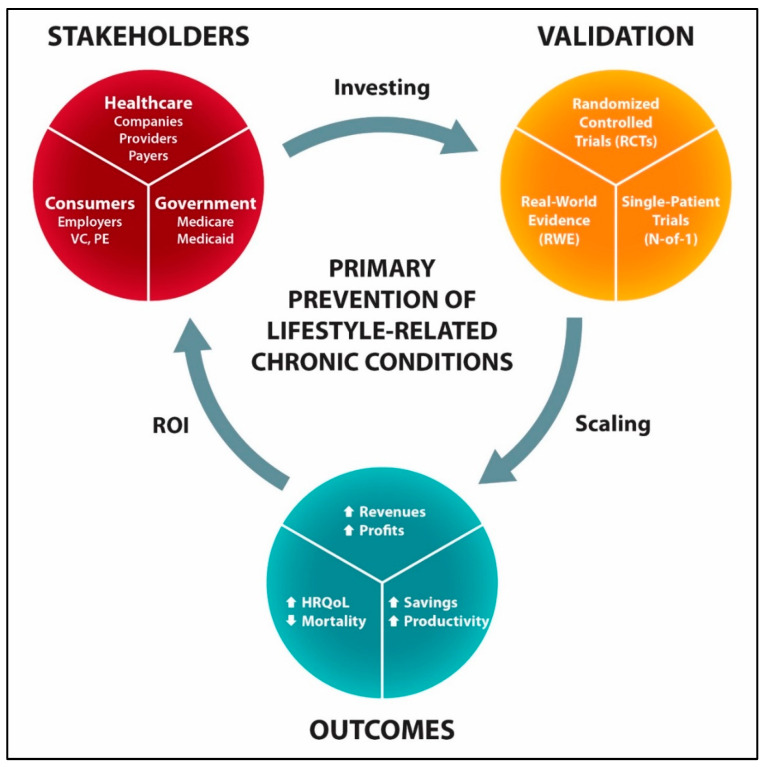
The Affordable Primary Prevention framework bridging key stakeholders, development, and return-on-investment (ROI). VC—venture capital; PE—private equity; HRQoL—health-related quality of life.

Figure 6 illustrates the Affordable Primary Prevention (APP) framework to support the development and scaling PDURS-based solutions for the primary prevention of diabetes, CVDs, and other lifestyle-related chronic conditions. Advancing the affordable primary prevention technology would include the following: (1) development of interoperable digital health platform, (2) long-term clinical validation and the regulatory approval, and (3) launching, marketing, and scaling. In addition to reduced mortality and morbidity rates, the projected APP outcomes would likely include the following: (1) APP sales revenues, (2) savings for payers, (3) savings and increased productivity for employers, and (4) profits for investors.

The following aspects may appeal to multiple stakeholders for investing in the affordable primary prevention:

- *Reduced development risk*, since the relevant existing health technologies (digital health platforms and metformin) are backed by real-world evidence regarding their effectiveness and safety.
- *Lower development costs*, as compared to an average cost for developing a new drug treatment, currently ranging estimates from USD 170–880 million (2018 dollars) to USD 944–2826 million (2019 dollars) [161,162].
- *Speed to market*, since digital health technologies offer early revenue generation as compared to traditional drug-based development.
- *Early market share* favors growing long-term relationships with consumers and commercial partnerships that support wellness and lifestyle medicine (e.g., wearables).
- *Low-cost prevention* mitigates socioeconomic barriers to care, enabling adoption through private- and government-sponsored programs at scale.
- *Decreasing profit margins for* payers and healthcare systems from increasing utilization of costly GLP1RA-based drugs and ultra-premium specialty treatments, e.g., CRISPR gene-editing and cell-based therapeutics.
- *The cost-related barriers of GLP-1 based drugs* even after the loss of exclusivity in 2032, assuming 74% discount for generic drug prices [163].
- *Obstacles of the government-based diabetes prevention program* including overwhelming requirements, challenges in receiving Medicare designation and reimbursements, insufficient payments for the Medicare Advantage plans, and others [164].

Investing in the innovation of health technologies is driven by return-on-investment (ROI) metrics that are determined by a combination of a market size and costs of medical interventions. Because the healthcare reimbursement structure disproportionately compensates providers for treating rather than preventing diseases, a market for the primary prevention of lifestyle-related chronic diseases has been largely overlooked and untapped. Value proposition of low-cost digital solutions for the primary prevention could be attractive for more traditional venture capital (VC) and private equity (PE) investment groups, when a Serviceable Obtainable Market (SOM) closely matches a Total Addressable Market (TAM). With estimated 98 million Americans living with prediabetes, 120 million with hypertension, and 100 million with obesity, TAM and SAM for the primary prevention can be substantial, assuming a buy-in from a majority of stakeholders. For example, digital prevention programs can currently range USD 30–60 per member per month (PMPM), yielding USD 360–720 per user/year (based on typical employer/payer contracts). Assuming low-cost solutions that could range USD 10–15 PMPM (for digital-first) to USD 20–30 PMPM (for metformin-enhanced DTx), even 10–20% market penetration could generate annual revenues exceeding USD 1 billion. Thus, potential revenues from selling primary prevention technologies and potential long-term savings (for the government, payers, and employers) can create sufficient incentives to invest in low-cost and low-development-risk primary prevention technologies.

There are many examples where VC funding has advanced the development of digital health technologies for cardiovascular diseases and diabetes [165,166]. Health systems are also recognized as investors to drive innovation of digital health [167]. There are nonprofit healthcare systems in the US that also have their own investment operations for healthcare innovation and transformation (Table S1). Herein, we suggest that collective efforts of venture funds from Kaiser Permanente, Intermountain Health, Cleveland Clinic, Ascension Health, and others could support development, clinical validation, real-world evidence (RWE), and adoption of digital health platforms intended for the primary prevention of cardiometabolic conditions. Commercial payers also have associated venture funds, e.g., Cigna Ventures, CVS Health Ventures, and others, advancing the digital transformation of healthcare (Table S2). Investing in primary prevention may appeal to wealthy individuals who already invest in healthcare and look to expand their social impact (Table S3). One example of such individuals is Mark Cuban, who has been transforming pharmacy care through his company Mark Cuban Drug Plus Cost company [168,169]. In addition to traditional venture capital (VC) groups, private equity (PE) funding plays an increasing role in healthcare [170]. While PE funding is mostly focused on asset management related to hospitals and physician groups and clinical networks, there are increasing investment activities towards digital healthcare transformation and precision medicine (Table S3). The perspective of “owning the market for the primary prevention of lifestyle-related chronic conditions” may appeal to PE groups, such as Carlyle Group, KKR, WCAS, TPG Capital, Blackstone Group, and others, that already invest in the USD 5 trillion healthcare industry, where chronic conditions dominate. Another possible strategy for investing in the APP framework includes public–private partnerships (PPPs) comprising key stakeholders ranging from nonprofit and commercial payers, employers, and investors (VC and PE groups) to state and federal funding agencies. Such a unified PPP could pool risk, standardize benefits, and fund DTx and metformin-enhanced DTx solutions at a scale that employer-based plans and fragmented healthcare cannot achieve. By aligning incentives across government, payers, and industry, this single-payer entity would support long-term investment in the affordable primary prevention of lifestyle-related chronic conditions.

## 7. Limitations and Challenges

Herein, we describe judicious opportunities to develop digital health technology that can be used as a monotherapy or as PDURS-based combination with metformin to prevent cardiometabolic conditions. Such digital and drug + digital interventions would offer individuals and healthcare systems affordable solutions to avoid the costly management of diabetes and CVDs. There are many digital health technologies that are aligned with opportunities to provide precision prevention interventions that comprise lifestyle modification, behavior change, and health education. However, at the same time, there are major limitations and challenges to develop and scale metformin-enhanced DTx for the primary prevention of diabetes and CVDs. The most apparent challenges are interoperability, incorporation of DTx-based data into EHR workflow, reimbursement of digital health platforms, privacy, and cybersecurity.

*The knowledge and research-practice gaps*: Despite growing clinical evidence, long-term RCTs and real-world studies are needed to support the effectiveness and cost-effectiveness of metformin and DTx in the primary prevention of diabetes and cardiovascular diseases in diverse at-risk populations. This knowledge gap also applies to children and adolescents [171,172,173]. There is also a significant lag between medical research studies and clinical practice that applies to both pharmacotherapies and digital health technologies. Since research on digital interventions for precision prevention of lifestyle-related chronic conditions is still in its infancy, the relevant knowledge gap includes strong clinical and real-world evidence for their long-term effectiveness and cost-effectiveness. Developing PDURS-based interventions is also relatively new and requires rigorous clinical testing in order to support clinically meaningful benefits of software in the drug + digital combination therapies.

*Long-term engagement*: The low retention in the National DPP highlights real-world challenges in long-term engagement in lifestyle interventions, even when supported by digital health technologies [174,175,176]. Improving engagement in digital DPP using AI/ML-based technology includes personalized patient–provider communications [177]. A recent review on improving adherence to self-management of diabetes, CVDs, and other chronic conditions found that “motivational strategies, such as feedback, health literacy, reminders, and motivational messages, goal-setting, social interaction, gamification, and rewards can improve patient adherence to self-care behaviors” [178]. AI-driven health agents can optimize digital content, difficulty, pacing, and behavioral prompts to each user’s real-time needs, thereby improving adherence over long periods [179]. In addition, coupling DTx- and AI-based personalized coaching with wearables, such as activity trackers, may improve engagement in lifestyle modification; however, more research is needed in evaluating long-term retention in the primary prevention using digital health technologies [110].

*Regulatory limitations*: There are very few FDA-approved drugs for the primary prevention and intended for people who are at high risk for developing a specific chronic condition (e.g., statins for CVDs, raloxifene for osteoporosis, or tamoxifen for breast cancer). However, prediabetes and “being at risk for CVDs” are not recognized by the FDA as medical indications, and metformin is not approved for the prevention of diabetes in the US. Therefore, developing DTx and metformin-enhanced DTx for the primary prevention of diabetes and CVDs is currently hampered by an inability to receive FDA authorization for such indications. As such, digital health platforms delivering precision prevention for cardiometabolic conditions could be marketed under the FDA “reinforcement discretion” as interventions “supporting cardiometabolic health for people at risk for diabetes and CVDs”. Since sustained-release metformin is approved for people at risk for diabetes in United Kingdom, it is possible that metformin-enhanced DTx can be approved in markets outside the US.

*Environmental impact on health*: Even the most effective pharmacological and behavioral interventions are limited by environmental factors that can increase risks for chronic conditions or/and exacerbate disease symptom. For example, research studies show that outdoor and indoor air pollution can increase risks for diabetes and CVDs [180,181,182,183]. Perhaps less known are negative effects of noise pollution on cardiovascular health [184,185]. The AHA recognizes the importance of misaligned light exposure causing circadian rhythm disruption and leading to cardiometabolic conditions [186]. One possible solution to mitigate the harmful effects of environmental factors and improve digital therapy outcomes is by optimizing home spaces for indoor environmental quality [187,188].

*Competition with the commercial determinants of health*: Prevention through developing healthy habits at home is recognized as a long-term solution for people at risk of chronic conditions [189]. However, establishing healthy nutrition habits is often confronted by ultra-processed food advertisements, which are shown to increase food intake [190,191]. We recently highlighted a challenging issue that retail pharmacies in the US promote sugar-sweetened and alcoholic beverages, despite evidence on their effects on cardiometabolic morbidities and cancer [136]. Promotion of sugar-sweetened beverages also happens in educational institutions that are associated with academic healthcare systems (Figure S1), undermining the promotion of healthy nutrition to prevent diabetes and heart attacks. These examples illustrate real-world challenges for people at risk for cardiometabolic conditions, even when using APP technology.

## 8. Conclusions

Integrating healthy behaviors with antidiabetic therapies is essential for financially sustainable prevention of cardiometabolic conditions. While GLP-1-based drugs offer promising solutions for managing cardiometabolic conditions, metformin-enhanced digital interventions offer meaningful potential for delivering affordable primary prevention of diabetes and CVDs. This new frontier in precision preventive medicine could bring a commonly used generic prescription drug back to mainstream healthcare, hence mitigating healthcare spending associated with high-cost GLP-1-based drugs. By integrating PDURS with metformin, healthcare providers can combine two mainstays of therapy, metformin and digital health interventions, into longitudinal preventive care for lifestyle-related chronic conditions. While there are diverse challenges and limitations to advance the Affordable Primary Prevention, the potential of metformin-enhanced DTx to reduce the financial burden of diabetes and CVDs may be a sufficient incentive to advance such approach. We hope that this perspective article will catalyze a dialog among all stakeholders about short- and long-term prospects of using the PDURS framework to innovate preventive medicine.

## Figures and Tables

**Table 1:** Comparing properties of metformin and GLP-1 based drugs in the primary prevention of cardiometabolic conditions.

Properties	Metformin	GLP1RA-Based Drugs
Mechanism of action	Pleiotropic: suppression of hepatic gluconeogenesis through activation of the AMPK pathway; enhancing insulin secretion; increasing GLP-1 release; reducing the DPP4 activity; anti-inflammatory [59,60,61,62].	Direct activation of GLP-1 receptors resulting in the increased insulin release, or a dual activation of GLP1/GIP receptors leading to decreased hunger and increased satiety; anti-inflammatory [63,64,65].
Prevention of diabetes	Lowers progression from prediabetes to T2DM by 17–31% compared to placebo. Metformin and lifestyle interventions reduce T2DM incidence by an additional 17% compared to lifestyle alone [66,67,68,69].	Lowers progression from prediabetes to T2DM by ~30–60% [70,71,72].
Prevention of MACE/CVDs	In comparison to placebo/no therapy, metformin decreases the risk of cardiovascular events and MACE [56,73,74,75,76].	In comparison to placebo/no therapy, GLP1RAs can reduce MACE by ~12–14% compared to placebo [42,77,78,79,80].
Weight loss	Modest effect on weight loss. In patients with T2DM, up to a 5% total body weight loss can be observed [55]. In patients without T2DM, compared to placebo, metformin can reduce BMI by 2.63% on average.	Reducing total body weight by ~5–15% depending on the medication itself, with liraglutide 3.0 mg leading to 5–8% body weight reduction and semaglutide 2.4 mg leading to 5 to over 15% body weight reduction [81,82].
Safety concerns	Lactic acidosis (very rare); vitamin B12 deficiency [83,84,85].	GI adverse events (nausea, vomiting, diarrhea, constipation) [86,87].
Monthly costs	USD 5.55 ^a^, or 6.09 ^b^	USD 500 ^c^

AMPK—AMP activated protein kinase; CVDs—cardiovascular diseases; DPP4—dipeptidyl peptidase-4; GIP—glucose-dependent insulinotropic polypeptide; Glucagon-like peptide; MACE—major adverse cardiovascular events. ^a^ Based on pricing 30-count 850 mg tablets from Mark Cuban Cost Plus Drugs pharmacy (as of October 2025). ^b^ Based on pricing 30-count 750 mg extended-release tablets from Mark Cuban Cost Plus Drugs pharmacy (as of October 2025). ^c^ Based on self-pay pricing of Ozempic in Costco (as of October 2025).

**Table 2:** Examples of digital health technologies supporting the prevention and management of diabetes and cardiovascular conditions.

Brand/ Supplier	Indication	Content	Website Listing Clinical Evidence
WellDoc, Bluestar	Diabetes (type 1 and 2), prediabetes, hypertension, heart failure, obesity and weight management	Real-time digital coaching; integration with CGMs; mental health tools; nutrition and exercise guides; tracking blood pressure, cholesterol, physical activity, calorie intake, and weight	www.welldoc.com (accessed 3 December 2025)
Dario Health	Diabetes, prediabetes, hypertension, weight management	Personalized and motivational coaching; virtual care; blood glucose and blood pressure monitoring	www.dariohealth.com (accessed 3 December 2025)
Omada Health	Prediabetes, diabetes, hypertension, weight management	Virtual care; medication management; CGM; remote monitoring; behavior change; health coaching and education	www.omadahealth.com (accessed 3 December 2025)
Dexcom G7, Stelo	Prediabetes, diabetes	CGM; detecting and reporting blood glucose spikes; logging meals, sleep, and physical activities; personalized insights; health education	www.dexcom.com www.stelo.com (accessed 3 December 2025)
Hello Heart	Hypertension	Tracking blood pressure, cholesterol, and medications; personalized health coaching and education	www.helloheart.com (accessed 3 December 2025)
AliveCor	Cardiovascular conditions	EKG and blood pressure monitoring; medication management	www.alivecor.com (accessed 3 December 2025)
Heart and Stroke Helper app	Stroke survivors	Self-management; tracking lifestyle habits; medication management; health education	Not available

**Table 3:** Clinical manifestations in people at risk and those with type 2 diabetes and cardiovascular conditions.

*Disease*	At Risk	Pre-Chronic Disease	Diagnosed Chronic Disease
Type 2 Diabetes Mellitus	>45 years NAFLD ^a^ Physical inactivity Overweight or obese	A1c = 5.7–6.4% FPG ^b^ = 100–125 mg/dL OGTT ^c^ = Oral 140–199 mg/dL	A1c > 6.5% FPG ^b^ > 126 mg/dL OGTT ^c^ > 199 mg/dL
Coronary Artery Disease	Existing T2DM Obesity Hypertension Hyperlipidemia		Diagnostic Tests: Electrocardiogram Echocardiogram Exercise stress test Chest X-ray Cardiac catheterization Coronary angiogram Coronary artery calcium scan
Hypertension	Physical inactivity Alcohol overuse Tobacco use Hereditary [85]	SBP ^d^ = 120–129 mmHg AND DBP ^e^ < 80 mmHg	SBP ^d^ > 130 mmHg OR DBP ^e^ > 80 mmHg
Heart Failure	Stage A: At Risk for heart failure	Stage B: Pre-Heart Failure	Stage C: Symptomatic Heart Failure Stage D: Advanced Heart Failure

^a^—NAFLD: Non-alcoholic fatty liver disease; ^b^—FPG: Fasting plasma glucose; ^c^—OGTT: Oral glucose tolerance test; ^d^—SBP: Systolic blood pressure; ^e^—DBP: Diastolic blood pressure.

## Data Availability

No new data were created or analyzed in this study. Data sharing is not applicable to this article.

## Supplementary Materials

The following supporting information can be downloaded at https://www.mdpi.com/article/10.3390/healthcare13243220/s1. Figure S1: A real-world example of the Commercial Determinants of Health that undermines prevention of diabetes and CVDs; Table S1: Selected examples of nonprofit healthcare systems in the United States that have venture groups. These healthcare systems and their venture arms are potential stakeholders in funding the Affordable Primary Prevention technologies; Table S2: Selected examples of commercial payers in the United States that have venture groups, as potential stakeholders in funding the Affordable Primary Prevention technologies; Table S3: Examples of wealthy individuals living in the US and investing in healthcare innovations.
