# Supplementary material for: Metformin-Enhanced Digital Therapeutics for the Affordable Primary Prevention of Diabetes and Cardiovascular Diseases: Advancing Low-Cost Solutions for Lifestyle-Related Chronic Disorders
Source: Healthcare (Basel). 2025 Dec 9;13(24):3220. doi: 10.3390/healthcare13243220 (PMC12732773; doi:10.3390/healthcare13243220)

Supplementary Materials

Metformin-Enhanced Digital Therapeutics for the Affordable Primary Prevention of Diabetes and Cardiovascular Diseases: Advancing Low-Cost Solutions for Lifestyle-Related Chronic Diseases

Brian Farley, Emi Radetich, Joseph DAlessandro, Grzegorz Bulaj

Table S1. Selected examples of nonprofit healthcare systems in the United States that have venture groups. These healthcare systems and their venture arms are potential stakeholders in funding the Affordable Primary Prevention technologies.

Healthcare System	Venture Arm	Examples of existing investments
Ascension	Ascension Ventures	Elation Health - EHR platform
Cleveland Clinic	Cleveland Clinic Ventures	Xealth – digital health benchmarking platform
Intermountain Health	Intermountain Ventures	Vori Health – virtual care
Kaiser Permanente	Kaiser Permanente Ventures	iRhythm – cardiac monitoring
Mass General Brigham	Mass General Brigham Ventures	Codametrix – medical coding platform

Table S2. Selected examples of commercial payers in the United States that have venture groups, as potential stakeholders in funding the Affordable Primary Prevention technologies.

Payer	Venture Arm	Notes
Cigna Corporation	Cigna Ventures	Invested in Omada Health, a digital health technology for managing chronic conditions, https://cignaventures.com/
Aetna	CVS Health Ventures	Invested in Amalgam, an AI-based platform to create digital health solutions, https://www.cvshealthventures.com/
Blue Cross Blue Shield	Blue Venture Fund	Invested in HeartFlow, a digital platfrom for coronary care, https://blueventurefund.com/

Table S3. Examples of wealthy individuals living in the US and investing in healthcare innovations.

Individuals	Examples of investments
Miguel B Fernandez	Physicians Healthcare Plans, CAC Medical Centers, Prescribit Rx, CarePlus Health Plans, Navarro Pharmacies, Medical Specialties Distributors, Hospitalists of America, ADI Dental, NutriForce, Simply Healthcare Plans, Acorn Health, Affinity Hospice, Strive Dental and Carisk Partners
Mark Cuban	Mark Cuban Cost Plus Drug Company
Bill Gates	Through the Bill & Melinda Gates Foundation's Strategic Investment Fund: BioNTech, Vendata Biosciences, Exscientia
Reed Jobs	Yosemite, Cancer-focused venture investing in Turquoise Health
Jeff Bezos	Through Bezos expeditions, invested in Mindstrong Health, Altos Labs, Grail Inc
James Breyer	Through Breyer Capital venture firm, invested in Atropos Health, SandboxAQ, Iterative Scopes, and Soley Therapeutics, Xaira, Paige AI
Elon Musk	Neuralink
Mark Zuckerberg	Through the Chan-Zuckerberg Initiative ventures, invested in Biohub, Citizen Health

Figure S1. A real-world example of the Commercial Determinants of Health that undermines prevention of diabetes and CVDs. Excessive consumption of sugar-sweetened beverages is associated with increasing risks for cardiometabolic morbidity and mortality (references summarized in Bulaj et al, Pharmacy 2025, https://www.mdpi.com/2226-4787/12/4/107). Shown is an example of promoting sugar-sweetened beverages as official refreshments of an academic institution that also has an academic medical center. Other universities that have the official partnership with Pepsico include University of Washington, University of Illinois Chicago, City University of New York, UC Davis and UC Berkeley.

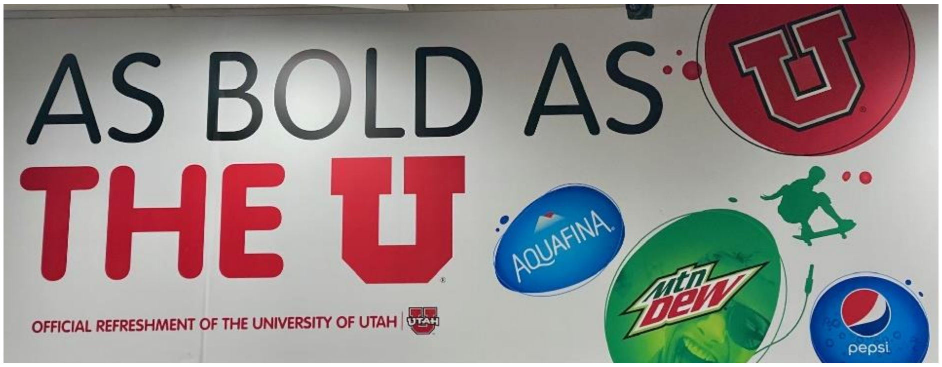

Supplement: Supplementary file 1 [file healthcare-13-03220-s001.zip › healthcare-3989964-supplementary.pdf]
